# Evaluating Conservative Versus Surgical Management Strategies in Omental Infarction: A Case Report and Literature Review

**DOI:** 10.1155/cris/6050351

**Published:** 2025-06-10

**Authors:** Yuki Julius Ng, Yee Siew Lim, Shivadeva Selvamani, Yew Wen Chieng

**Affiliations:** ^1^Department of General Surgery, Sarawak General Hospital, Kuching, Sarawak, Malaysia; ^2^Department of General Surgery, International Medical University, Kuala Lumpur, Malaysia

**Keywords:** omental infarct, omental torsion, pedicle sign, whirl sign

## Abstract

Omental infarction was first described in 1896 mimics other causes of acute abdomen. Improved imaging modalities such as ultrasound and CT scans, have enhanced preoperative diagnosis with conservative management emerging as a treatment option. We report the case of a 51-year-old man presenting with epigastric pain migrating to the right iliac fossa, fever, nausea and anorexia. Examination revealed a stable patient with a right lumbar mass (5 cm × 6 cm) and rebound tenderness. CT imaging identified fat stranding near the ascending colon and hepatic flexure (6 cm ×10 cm ×10 cm) with peritoneal thickening. He underwent exploratory laparotomy, omentectomy and peritoneal washout, which revealed an infarcted omentum (8 cm × 8 cm) and 200 ml of haemoserous fluid. The patient recovered well postoperatively. A systematic search of the literature identified 237 articles reporting 479 cases of omental infarction, with clinical data extracted for analysis. Male predominance was observed (2:1) and 326 patients (68.1%) underwent surgical intervention. Conservative management was successful in 121 patients (25.3%), while 32 (6.7%) required surgery following failed conservative treatment. Among those managed surgically, the most common preoperative diagnosis was appendicitis. CT imaging was performed in 245 cases (51.1%), of which 103 (42.0%) within this group were successfully managed conservatively, while 26 (10.6%) required surgical intervention after conservative failure. Omental torsion was diagnosed preoperatively in 220 patients (45.9%); in this subset, 21 (9.5%) underwent surgery after failed conservative management and only 5 (2.3%) were successfully treated conservatively. While omental infarction can often be managed conservatively, surgery remains a key treatment for intractable pain or omental torsion, where conservative management failure rates are high.

## 1. Introduction

Omental infarction was first described in 1896 by Bush [1] and further reported by many authors. Since then, there is a lack of consensus on the treatment of the condition. There is a polarised view on the management of conservative treatment and operative management [2–4]. Some literature claims there are about 250 cases reported globally; however, this number is not consistent with published literature [5]. We reported a case of omental infarct and performed a systematic literature review to provide the management of omental infarct, how clinicians decide on the management globally and the number of documented cases of omental infarct. We also attempted to provide the total number of reported omental infarctions and understand the distributions among genders, age and analyse the management worldwide. Some literature suggested the management of omental infarction should include conservative management if the patient is clinically well, but some suggested surgery would reduce the duration of hospital stay and significantly reduce the risk of other complications that are associated with hospital admissions. Therefore, to find out which management provides the best outcome, we performed a literature review aimed to design a flowchart that could guide clinical decisions of omental infarction and torsion.

## 2. Case

Our patient is a 51-year-old man with underlying hypertension, hyperlipidaemia, diabetes mellitus and human immunodeficiency virus, who presented with abdominal pain for 4 days over the right iliac fossa. It initially started over the epigastric region and migrated over to the right iliac fossa. He also complained of fever for 1 day, nausea and loss of appetite. His observations were stable. Physical examination showed a right lumbar mass of about 5 cm × 6 cm, tender and firm. Rebound tenderness was positive, but the Rovsing sign was negative.

## 3. Investigation

**Figure 1:**
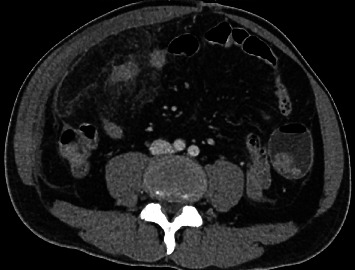
CT axial.

**Figure 2:**
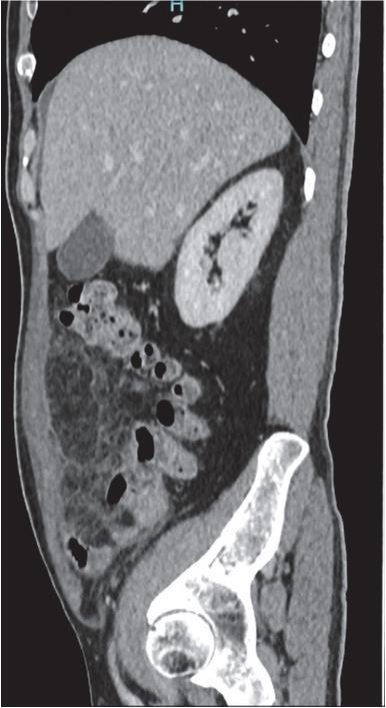
CT sagittal view. CT abdomen and pelvis. Increased fat stranding of omentum along anterior aspect of ascending colon and inferior to hepatic flexure measuring 6 cm × 10 cm × 10 cm (A P × W × CC) with adjacent peritoneal thickening and enhancement with evidence of thrombosed mesenteric vessels.

Blood investigation for coagulation profile, biochemistry, renal function test, liver function, amylase and blood gases were all within normal range. His total white cells were 13,700/mm_3_. CT scan showed an area of increased fat stranding of omentum along the anterior aspect of the ascending colon and inferior hepatic flexure, measuring 6 cm × 10 cm × 10 cm associated with adjacent peritoneal thickening (Figures 1 and 2). Findings suggest omental infarction.

**Figure 3:**
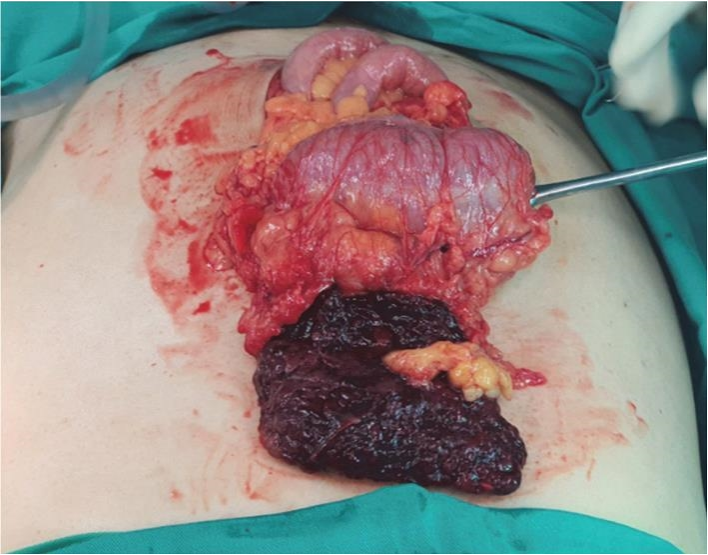
Area of omental infarct about 8 cm × 8 cm.

Our patient underwent exploratory laparotomy, omentectomy and peritoneal washout. Intraoperatively, there was 200 mls of haemoserous peritoneal fluid upon entry. The omentum was infarcted 8 cm × 8 cm, adhered to the ileum and sigmoid colon (Figure 3). An intraabdominal drain was placed. Postoperatively, our patient recovered well and was discharged after the reduction of the pelvic drain.

## 4. Follow-Up

The patient was followed up at the outpatient surgical clinic without any complications.

## 5. Discussion

### 5.1. Literature Search

A literature search was done from 1986 until 2024 on PubMed. We searched with the titles and abstract (tiab) function with Omentum AND Infarction, Omentum torsion. We initially found 9883 articles, we then excluded the duplicates and uploaded to RAYYAN and we included patients of all ages, gender and diagnosis of omental infarction and omental torsion. We excluded omental infarction from a hernia and epiploic appendagitis. This exclusion was done as omental infarct secondary to hernia is usually managed by operative repair and epiploic appendagitis should be managed conservatively. From this initial search, we found only 126 articles. We further searched for Grey literature from Google Scholars and references of the literature that has a literature review and other published articles. We further found 220 articles. We then further excluded the duplicates and then, we extracted data which are summarised in (supporting information). We excluded 32 articles from the analysis because they were either not accessible, not archived or the journals were disbanded.

**Figure 4:**
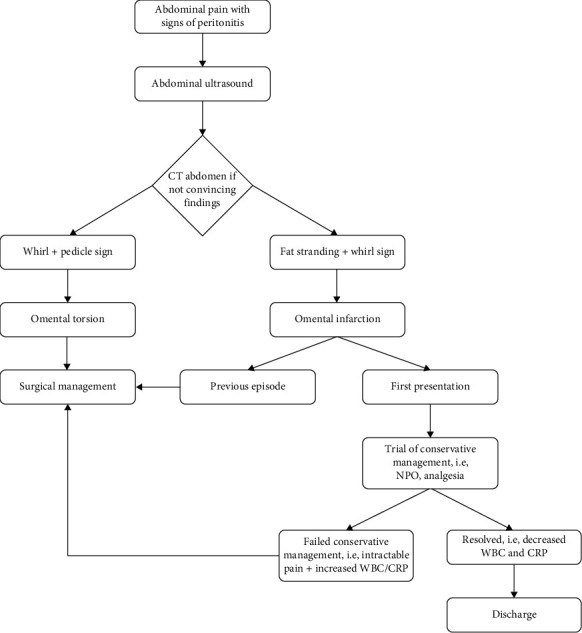
Flow chart of clinical decision making for omental infarction.

We found a total of 479 cases from 236 articles. The condition showed a higher prevalence in males with a 2:1 ratio. There were three times more adults over 18 years old who presented with omental infarction. Not all patients were investigated with bloods or scans. Out of 368 (76.8%) that had blood investigation, 179 patients had normal white cell count and 189 patients with raised white cell count. There were 130 (27.1%) patients tested with CRP and 104 (80%) of them were raised. Three hundred and forty nine (72.9%) patients were not investigated with CRP. CT scans were done to 245 (51.1%) patients and was considered the most reliable diagnostic tool, followed by ultrasound that was done in 209 (43.6%) cases. While 153 cases were initially managed conservatively, 32 required surgical intervention due to treatment failure. Overall, surgical management was performed in 358 cases, including those that failed conservative management. Intraoperative findings frequently revealed omental torsion with necrosis, serosanguineous fluid and mesenteric lymphadenopathy, which was histologically confirmed with inflammation, venous congestion and thrombosis. Omental torsion was diagnosed in 220 patients (45.9%); in this group, 194 (88.2%) patients were treated surgically, 21 (9.5%) underwent surgery after failed conservative management and only five (2.3%) were successfully treated conservatively. Out of 32 patients who failed conservative treatment, 65.6% of them were diagnosed as omental torsion. The collated data extracted from the articles provided clinical decisions made by clinicians globally. These decisions were carefully represented as a flowchart for the management of omental infarction and omental torsion (Figure 4).

Clinically all patients reported in this search presented symptoms like acute appendicitis as well as biliary colic which was then investigated with abdominal ultrasound. This increases the diagnostic difficulties without imaging. Where centres did not use imaging such as CT scan or ultrasound scan because of local policies, they would often find a normal appendix or gall bladder and would perform omentectomy with or without taking the appendix out. Ultrasound will usually show a negative finding of the two common working diagnoses which helps clinicians to decide for CT scans [3, 6, 7]. CT will then provide a definitive diagnosis of omental infarction with radiological findings of fat stranding and or whirl sign as demonstrated in Figures 1 and 2 [3, 7, 8]. Omental torsion could additionally be diagnosed when pedicle sign is present [9–12]. With the definitive diagnosis, the decision to operate becomes unclear as there is no consensus between clinicians whether to operate or not [2–4]. Many patients have been successfully treated with conservative management which shifts the attention away from operative management [13, 14]. This, however, is not the case as some patients fail conservative management, and their intractable abdominal pain resolves with surgical resection of the omentum [2, 4, 15].

Conservative management should always be considered as up to 23.6% of cases reported from our literature search suggests that it is successful. Although a large majority of 76.4% of cases were successfully managed with surgical resection, the authors believe most surgeons globally opt to operate instead of a conservative approach. What was found to be interesting were the 32 patients that failed conservative management. About two-thirds of these patients have torted omentum (Table 1). Some cases of omental infarction even reported the omentum to be twisted up to 18 times [9]. In which case, it would be sensible to decide for surgery to remove the torted tissue causing ischaemia and intractable abdominal pain. Among those diagnosed with omental infarction without torsion, almost half of them were successfully treated conservatively. These patients presented with omental infarction for the first time (Table 1). Within this group, only 4.2% experienced failed conservative management which is reassuring considering the high success rate with conservative management. In our hospital, because our population of patients usually stay more than 2 h of commuting distance, our consultants have decided that surgery will provide a definitive management for our patient. The role of minimally invasive surgery can also benefit patients undergoing surgery for omental infarction in terms of scaring, postoperative pain management and days of hospital stay. Many cases from our literature search has performed them, but were not analysed as there were missing data from the reported cases. From the literature, most if not all patients were discharged within the same day of operation which includes both open and laparoscopic approach.

The clinical features of omental infarction are often similar to many causes of acute abdomen which makes it challenging to diagnose clinically. Although omental infarction is considered to be a rare cause of abdominal pain, a total of 479 cases were collated and it is probably under-reported in the literature.

## 6. Conclusion

Omental infarction can be treated conservatively with fluids, analgesia and antibiotics. However, if the symptoms worsen or if patients had previous episodes, surgery should be considered to treat the intractable pain. Patients who were diagnosed with omental torsion should undergo surgery because of the high failure rate of conservative treatment. The limitation of operating in patients with omental infarction would be the surgeon's confidence and experience in performing the surgery both open and minimally invasive approach.

## Figures and Tables

**Table 1:** Summary of collated clinical data.

Variable	*n* (%)
Gender	
Male	309 (64.5%)
Female	164 (34.2%)
Not reported	6 (1.3%)
Age	
<18	109 (22.8%)
>18	330 (68.9%)
Not reported	40 (8.3%)
White cell count (4000–11,000 mm^3^)	
Raised WBC	189 (39.5%)
Normal WBC	179 (37.4%)
Not reported	111 (23.1%)
C reactive protein (<5 mg (L))	
Raised	104 (21.7%)
Normal CRP	26 (5.4%)
Not reported	349 (72.9%)
Imaging	
Ultrasound abdomen	209 (43.6%)
CT scan	245 (51.1%)
Management	
Surgical management (initial management)	326 (68.0%)
Conservative management	121 (25.3%)
Failed conservative management (surgical management)	32 (6.7%)

## Data Availability

The data that support the findings of this study are available in the supporting information of this article.

## Supporting Information

Supporting Information A summarised collated data of omental infarction and their management.
